# Identification of Arenin, a Novel Kunitz-Like Polypeptide from the Skin Secretions of *Dryophytes arenicolor*

**DOI:** 10.3390/ijms19113644

**Published:** 2018-11-19

**Authors:** Jesús Hernández-Pérez, Aida Serra, Siu Kwan Sze, Patricia L. Conway, Jørgen Schlundt, Jorge Benavides

**Affiliations:** 1 School of Engineering and Science, Tecnologico de Monterrey, Av. Eugenio Garza Sada 2501 Sur, C.P. 64849 Monterrey, N.L., Mexico; a00799272@itesm.mx; 2 School of Biological Sciences, Nanyang Technological University, 60, Nanyang Drive, Singapore 637551, Singapore; aserramaq@ntu.edu.sg (A.S.); sksze@ntu.edu.sg (S.K.S.); 3 Nanyang Technological University Food Technology Centre (NAFTEC), Nanyang Technological University (NTU), Singapore 637459, Singapore; pconway@ntu.edu.sg (P.L.C.); jschlundt@ntu.edu.sg (J.S.); 4 School of Chemical and Biomedical Engineering, Nanyang Technological University (NTU), 62 Nanyang Drive, Singapore 637459, Singapore

**Keywords:** amphibians, skin secretions, *Dryophytes arenicolor*, Kunitz-like polypeptide, cytotoxicity

## Abstract

Amphibian skin secretions are enriched with complex cocktails of bioactive molecules such as proteins, peptides, biogenic amines, alkaloids guanidine derivatives, steroids and other minor components spanning a wide spectrum of pharmacological actions exploited for centuries in folk medicine. This study presents evidence on the protein profile of the skin secretions of the canyon tree frog, *Dryophytes arenicolor*. At the same time, it presents the reverse-phase liquid chromatography isolation, mass spectrometry characterization and identification at mRNA level of a novel 58 amino acids Kunitz-like polypeptide from the skin secretions of *Dryophytes arenicolor*, arenin. Cell viability assays performed on HDFa, CaCo2 and MCF7 cells cultured with different concentrations of arenin showed a discrete effect at low concentrations (2, 4, 8 and 16 µg/mL) suggesting a multi-target interaction in a hormetic-like dose-response. Further work is required to investigate the mechanisms underlying the variable effect on cell viability produced by different concentrations of arenin.

## 1. Introduction

Amphibians were the first group of vertebrates to make the transition from water to land [1] and over millions of years have been developing a multifunctional skin that is morphologically and biochemically adapted to accomplish both general physiological and more specific survival roles, such as chemical defense against predators [2,3].

Skin secretions synthesized and stored in highly specialized granular glands contain complex cocktails of bioactive molecules such as proteins, peptides, biogenic amines, alkaloids guanidine derivatives, steroids and other minor components spanning a wide spectrum of pharmacological actions exploited for centuries in folk medicine [4,5,6,7].

Due to vast amphibian biodiversity, diverse applications and an enormous array of bioactive molecules that have not been described yet, frog and toad (Anurans) skin secretions continue drawing attention as source of novel biologically active molecules [4,8]. Although peptides of 10 to 48 residues long comprise the majority of biomolecules described in literature [8], a considerable number of species from distinct families have been described to lack peptides in their skin secretions [9,10,11,12].

Hylidae is one of the largest families of anurans with over 870 species recognized and is considered a rich source of amphibian bioactive peptides. The vast majority of the bioactive peptides and proteins described belong to the subfamilies Pelodryadinae and Phyllomedusinae even though the subfamily Hylinae has the widest distribution and the most species [13,14]. From the hylinae subfamily, the *Hyla* genus, recently divided into Old World (*Hyla*) and New World (*Dryophytes*) subgenera [15], comprises 35 species from which antimicrobial peptides, neuropeptides, wound-healing peptides, antinociception peptides, protease inhibitor peptides, tryptophyllins and caeruleins have been described [8].

Based on the evidence of the presence of bioactive peptides in amphibian skin secretions and taking advantage of the enormous biodiversity in Mexico, we selected a member of the Hylidae family that has been mentioned in ethnopharmacological studies as an ingredient used in Mexican Traditional Medicine (MTM) practices [16,17], to study its skin secretions. Here, we present the first report about the characterization of the skin secretion of *Dryophytes arenicolor*, formerly known as *Hyla arenicolor* [15], a frog with an atypical skin secretion profile due to the absence of low molecular weight peptides and a preliminary activity characterization of its most abundant fraction.

## 2. Results

### 2.1. Collection and Identification of D. arenicolor

All specimens utilized in this study were collected in one night at the same location according to their call and morphological characteristics such as size, skin and leg color. Further molecular identification was carried out as described in the collection and identification methodology section. Polymerase chain reaction (PCR) products (GenBank: MG554648) amplified from total DNA extracted from freeze-dried skin of collected frogs using MVZ-59^F^ and tRNAVal^R^ primers resulted in a 1012 bp fragment corresponding to partial sequences of 12S ribosomal RNA and tRNA-Val genes of *D. arenicolor*, formerly known as *Hyla arenicolor*, when searched against the nr/nt database from GenBank using local NCBI-BLASTn software (v2.7.1+).

### 2.2. Recovery of Skin Secretions Extract

Following the methodology described in the Recovery of the Skin Secretion Extract (SSE) section, average total soluble protein recovered in SSE was 90 ± 15 µg per gram body weight. None of the frogs tested showed any health complication after release from skin secretions recovery and norepinephrine stimulation.

### 2.3. Skin Secretion Extract (SSE) Characterization by 2D Polyacrylamide Gel Electrophoresis (2D-PAGE) and Reverse-Phase High-Performance Liquid Chromatography (HPLC)

**Figure 1:**
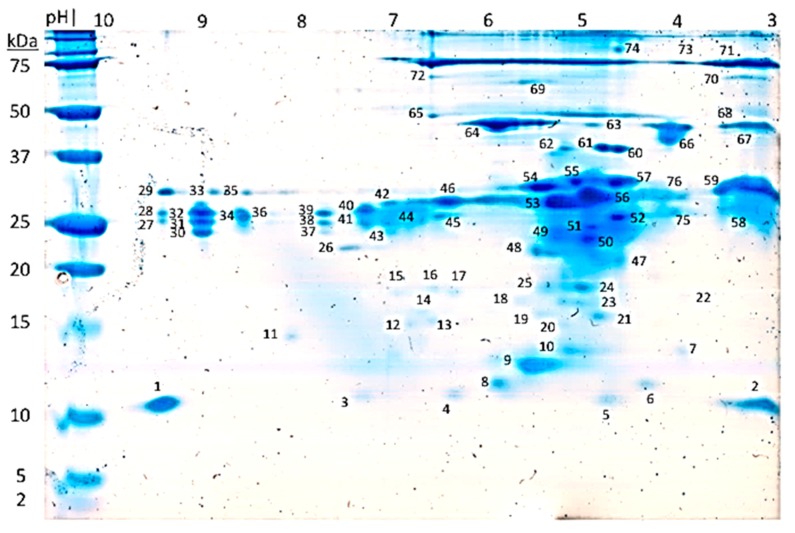
*D. arenicolor*’s skin secretions extract (SSE) Tricine-2D polyacrylamide gel electrophoresis (2D-PAGE). 150 µg of SSE analyzed by Tricine-2D-PAGE yielded 76 spots stained with Colloidal Coomassie Blue.

According to the methodology described for the SSE characterization by 2D gel electrophoresis, 76 spots were stained (Figure 1), revealing the absence of detection of peptides or oligopeptides below 10 kDa, a low content of proteins between 10 and 20 kDa with very well defined spots at pH 3 and pH 7 around 10 kDa; a high content of proteins between 20 to 37 kDa with a higher density between pH 4 and pH 6; and a low content of proteins between 37 and 75 kDa with all of the proteins between pH 3 and 7.

**Figure 2:**
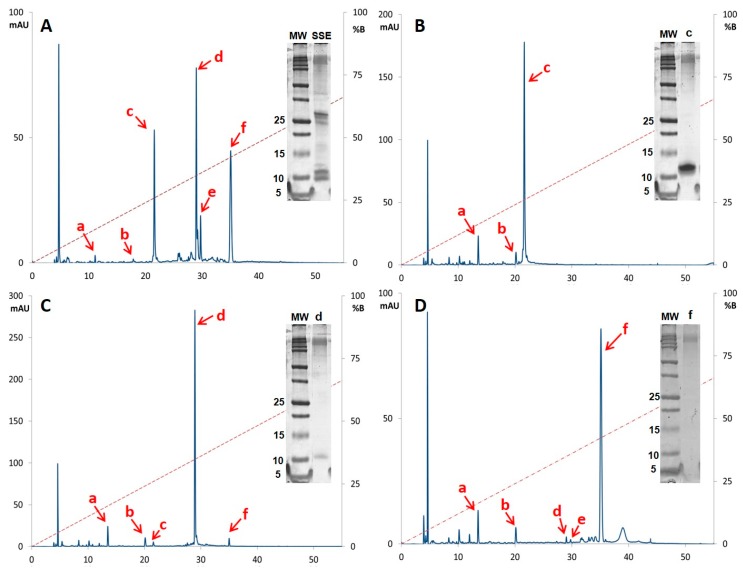
Analytical reverse-phase chromatograms and Tricine sodium dodecyl sulfate (SDS)-PAGE of the more abundant fractions recovered after SSE semi-preparative reversed-phase high-performance liquid chromatography (RP-HPLC) fractionation. (**A**) SSE with recovered fractions indicated by letters a, b, c, d, e and f. (**B**) Fraction c had a retention time of 22.3 min and an apparent molecular weight around 12 kDa. (**C**) Fraction d showed correlation with the 214 nm signal at 30.2 min and an apparent molecular weight around 11 kDa. (**D**) Fraction f showed 214 nm signal at 36.2 min, however it was not possible to correlate this fraction with any Tricine-PAGE band. Red dotted line represents the mobile phase composition for elution in relation to solvent B. *M*_w_: Molecular Weight marker; mAU: milli-Absorbance Units.

Following the reversed-phase high-performance liquid chromatography (RP-HPLC) method described in the Materials and Methods section, analysis of SSE (Figure 2A) revealed more than 30 peaks when the diode array detector was set to 214 nm for detection of peptide bond (–CONH–) [18]. Compounds with retention times at 22.3 min (Fraction c), 30.2 min (Fraction d) and 36.2 min (Fraction f) were the most abundant species across all specimens tested. Minor variations on the relative signal of these compounds between SSE of different individuals were observed, meanwhile the elution profile pattern was maintained reproducible along this study.

### 2.4. Protein Isolation for Activity Evaluation

Based on the RP-HPLC fractionation method described in the protein isolation section, 3 fractions eluting at 10.5 min (Fraction c), 13.6 min (Fraction d) and 22.1 min (Fraction f) were the most abundant species, promoting its purification. Fractions with the same retention time were pooled and analyzed through Tricine-polyacrylamide gel electrophoresis (PAGE) and RP-HPLC (Figure 2) employing the method described in the SSE characterization section.

RP-HPLC analysis revealed that fractions c, d and f corresponded to the 214 nm signals detected at 22.3 min (Figure 2B), 30.2 min (Figure 2C) and 36.2 min (Figure 2D) respectively. Tricine-PAGE showed the correlation between the purified fractions and their apparent molecular weight. Fractions c and d yielded the lowest bands between the molecular marker bands of 10 kDa and 15 kDa, where the band produced by fraction c resolved slightly above the band displayed by fraction d. Fraction f showed a strong 214 nm signal at 36.2 min in RP-HPLC analysis, however it was not possible to detect this fraction through Tricine-PAGE stained either with silver nitrate or colloidal Coomassie.

### 2.5. Cell Proliferation Inhibition by SSE and HPLC-Purified Fractions

**Figure 3:**
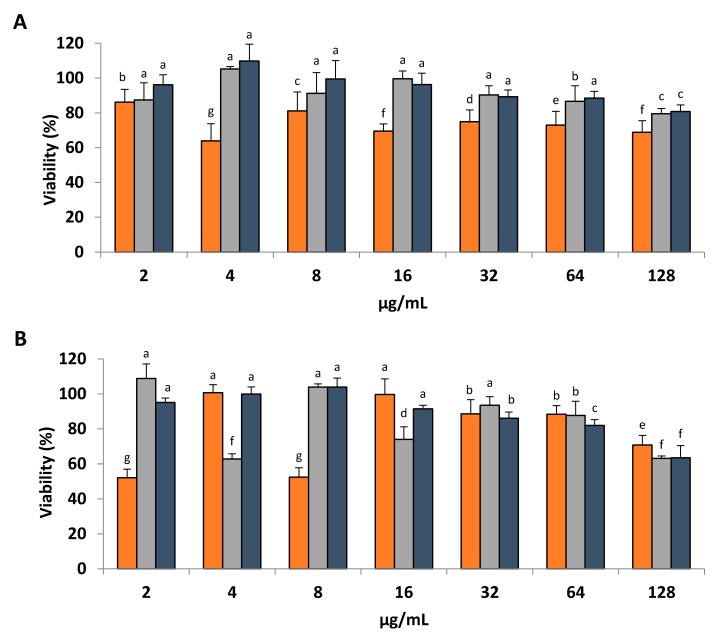
Cell proliferation assay. Viability of (■) HDFa, (■) CaCo-2 and (■) MCF7 cells cultured with different concentrations of (**A**) SSE and (**B**) HPLC Fraction c is presented as a percentage of the formazan signal recorded at 490 nm compared to untreated cells. According to the Tukey’s test performed, significantly different means are identified with different significance letters.

As described in the Materials and Methods section, in order to estimate proliferation of HDFa, CaCo2 and MCF7 cells in the presence of SSE and HPLC fractions at 2 µg/mL, 4 µg/mL, 8 µg/mL, 16 µg/mL, 32 µg/mL, 64 µg/mL and 128 µg/mL, a colorimetric method based on bioreduction of tetrazolium into formazan by cells was selected. Results in Figure 3 are presented as a viability percentage calculated in relation to untreated cells representing 100% viability.

Interestingly, and contrary to what we were expecting, a continuous dose-response relationship was not observed in any of the cell lines tested with both the SSE and the most abundant HPLC fraction (fraction c, Figure 2B). Cells treated with SSE showed a discontinuous trend (Figure 3A). HDFa cells cultured in the presence of SSE showed its highest viability of 86.12% ± 4.30 at 2 µg/mL, at 4 µg/mL cell viability decreased to its lowest value of 63.87% ± 5.71, then increased to 81.07% ± 6.36 at 8 µg/mL, dropped to 69.55% ± 2.35 at 16 µg/mL, increased at 32 µg/mL to 74.86% ± 3.94, decreased to 72.97% ± 4.58 at 64 µg/mL and continue decreasing to 68.82% ± 3.84 at 128 µg/mL.

Caco-2 cells treated with SSE at 2 µg/mL presented a viability of 87.45% ± 5.69 that increased to 105.25% ± 0.78 at 4 µg/mL, then decreased to 91.16% ± 6.95 at 8 µg/mL, increased to 99.61% ± 2.55 at 16 µg/mL, decreased to 90.36% ± 2.99 at 32 µg/mL, continued decreasing to 86.64% ± 5.17 at 64 µg/mL, and reached its lowest viability of 79.49% ± 1.73 at 128 µg/mL

The SSE effect on MCF7 cells was very similar to the response caused by HPLC fraction c also on MCF7 cells. At 2 µg/mL of SSE, MCF7 cell viability was 96.11% ± 3.36, at 4 µg/mL a 9.84% ± 5.55 surpass on the viability compared to untreated cells was observed, cell viability at 8 µg/mL decreased to 99.5% ± 6.06 and continued decreasing up to 80.80% ± 2.16 at 128 µg/mL.

HDFa cells treated with fraction c (Figure 3B) at 2 µg/mL and 8 µg/mL showed a significant reduction in viability, 52.1% ± 2.86 and 52.46% ± 3.08 respectively, while the viability of HDFa cells treated with 4 µg/mL of fraction c was almost the same as the viability of untreated cells. A continuous decline in HDFa cells viability was observed as fraction c concentration increased from 16 µg/mL to 128 µg/mL.

CaCo2 cells treated with 2 µg/mL of fraction c outperformed the viability of untreated cells by 8.8% ± 4.86, for 4 µg/mL viability dropped to 63.87% ± 5.71, at 8 µg/mL increased to 103.96% ± 1.05, dropped at 16 µg/mL to 73.95% ± 4.17, increased at 32 µg/mL to 93.47% ± 2.84, decreased to 87.72% ± 4.65 at 64 µg/mL and finally reached its lower viability of 63.09% ± 0.83 at 128 µg/mL.

A less discontinuous trend was observed for MCF7 cells treated with fraction c, although cells viability at 2 µg/mL was 95.09% ± 1.44, increased to 99.88% ± 2.39 at 4 µg/mL continued increasing to 103.96% ± 2.97 at 8 µg/mL, a tendency shift took place at 16 µg/mL with 91.46% ± 1.16 MCF7 cells viability and continued decreasing until 63.5% ± 2.16 at 128 µg/mL of fraction c.

### 2.6. Structural Characterization of Bioactive Fraction

**Figure 4:**
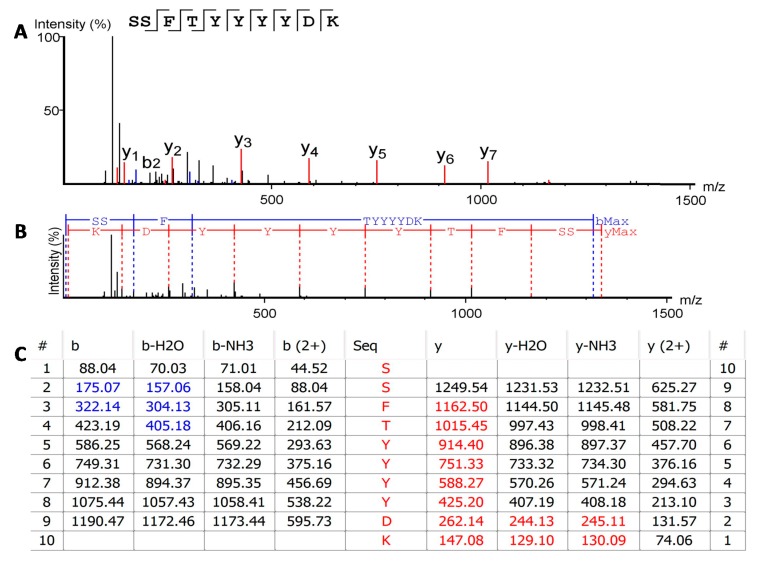
De novo sequence prediction of the most abundant trypsin-digested peptide from fraction c. (**A**) Annotated spectrum of peptide SSFTYYYDK showing identified b (blue signal) and y (red signal) ions. (**B**) Alignment of the spectrum with the fragment ions generated from the peptide, bMax: is the most intense peak in the spectrum corresponding to b ions series (blue uppercase letter and lines); yMax: is the most intense peak in the spectrum corresponding to y ions series (red uppercase letter and lines). (**C**) Predicted b and y ions match table with the calculated mass of possible fragment ions; spectrum matching b ions are highlighted in blue and spectrum matching y ions are highlighted in red, #: identifies the order of the ions identified in the spectrum according to each ion series.

Following the methodology described to elucidate the primary structure of the bioactive proteins in the SSE of *D. arenicolor*, several uncharacterized sequences were predicted using PEAKS software-assisted de novo sequencing combined with the use of a tailored database containing all anuran mRNA and Protein entries available in NCBI. From the de novo sequencing analysis of fraction c, tryptic-digested peptide sequence SSFTYYYYDK (Figure 4) was predicted with high average local confidence (ALC) (>95%). A similarity sequence analysis using BLASTp showed 80% identity with a protein named anntoxin, first described in the skin secretions of *Hyla annectans* [19] and later on *Hyla simplex* [20]. Other peptides with lower ALC and matching different regions of the anntoxins accounting for 79.31% of the full sequences were also predicted since the anntoxin region Y^36^RGXGGNGNRFK^47^ was not proposed by the de novo algorithm.

It was not possible to obtain any peptide with a high ALC from fractions d and f through the de novo sequencing strategy due to the reduced genomic and transcriptomic information available on anurans. Based on the predicted peptides, it is possible that fraction d is a protein which primary structure has none or low homology with any other anuran protein or peptide previously described. Meanwhile, due to its absorption at 214 nm, it is likely that fraction f is a peptide or even a protein, however this only would be confirmed through evidence at mRNA level.

### 2.7. Identified Protein cDNA Synthesis

**Figure 5:**
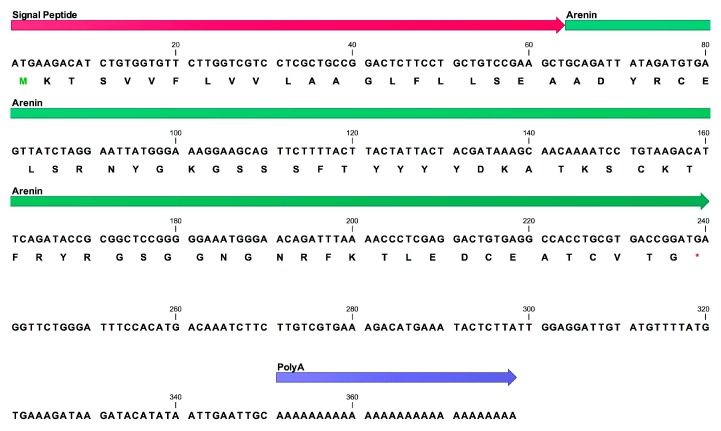
Full cDNA sequence of encoded arenin from *D. arenicolor* skin secretions. (■) Codified and translated signal peptide, (■) nucleotide and amino acid sequences of the mature polypeptide arenin, (■) Poly A signal. Green residue indicates translation initiation codon. Red asterisks indicates translation stop codon.

From the de novo sequencing of the most abundant trypsin-digested peptide from fraction c, SSFTYYYYDK, further BLASTp and partial homology correlation with the anntoxins from *H. annectans* and *H. simplex*, a degenerated 5′ primer, denominated RAnx1, was designed based on the amino acid sequence KTSVVFL from the signal peptide sequence of the previously characterized anntoxins. 3′ rapid amplification of cDNA ends (RACE) PCR reactions that employed the universal primer mix (UPM) as 3′ primer and degenerated RAnx1 as 5′ primer yielded a 378 bp product. BLASTn of this PCR product showed overall nucleotide sequence similarity of 87% between *H. annectans*’ anntoxin and the cDNA amplified with RAnx1 and UPM primers from the mRNA recovered from the skin secretions of *D. arenicolor*. The 378 bp PCR product encompasses signal peptide, mature peptide and poly A signal (Figure 5). Signal peptide was predicted just before the residue Ala^22^ using the SignalP 4.1 server and its nucleotide sequence showed 95% similarity with the signal peptide sequence of *H. annectans*’ anntoxin. Meanwhile, the 58 amino acids mature protein encoded by the PCR product showed a 93% identity with the anntoxin from *H. annectans* and 82% with the anntoxin S1 from *H. simplex.* Following amino acids were unique in the protein from the skin secretions of *D. arenicolor* when aligned with *H. annectans* and *H. simplex* anntoxins: Glu^6^ Ser^16^ Tyr^20^ and Lys^28^. Mature protein encoded by the cDNA generated from the skin secretions of *D. arenicolor* has been named by our research group as Arenin. The complete cDNA sequence was deposited in GenBank under the accession number MH898942.

Further BLASTp revealed that, as the anntoxins from *H. annectans* and *H. simplex*, arenin could have a Kunitz/Bovine pancreatic trypsin inhibitor (BPTI) domain, usually indicative of serine protease inhibitory activity. A possible trypsin interaction site *ku* [21] was also identified, comprising the residues Lys^13^Gly^14^Ser^15^Ser^16^Ser^17^Thr^19^.

### 2.8. Structural Modeling of Arenin

**Figure 6:**
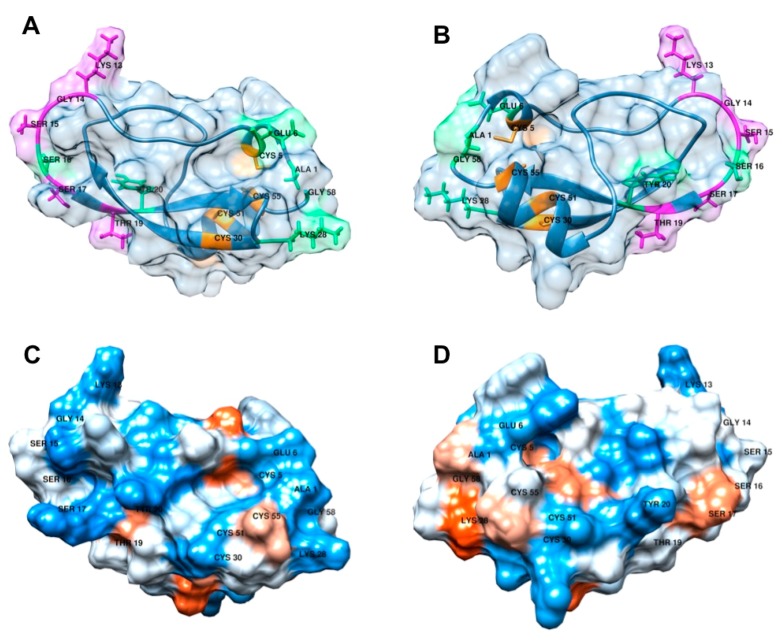
3D model of arenin from *D. arenicolor* skin secretions. (**A**) Front and (**B**) back ribbon model of arenin, Unique amino acids Glu^6^ Ser^16^ Tyr^20^ and Lys^28^ are highlighted in green, amino acids Lys^13^Gly^14^Ser^15^Ser^16^Ser^17^Thr^19^ possibly constitutes the trypsin interaction site *ku* and are highlighted in purple, amino acids Cys^5^-Cys^55^ and Cys^30^-Cys^51^ involved in the formation of disulfide bonds are highlighted in orange. (**C**) Front and (**D**) back polarity model prediction of arenin, blue zones represent polar residues, red zones non-polar residues and white zones neutral residues.

The 3D model of arenin (Figure 6) generated based on the solution structure of anntoxin from *H. annectans* suggests that arenin keeps a very similar structure with anntoxin maintaining the two disulfide bonds Cys^5^-Cys^55^ and Cys^30^-Cys^51^ due to the proximity between the sulfhydryl groups of each pair of cysteines, 2 alpha-helix motifs Tyr^3^ to Glu^6^ and Leu^48^ to Cys^55^ and a twisted 2-stranded antiparallel beta sheets Phe^18^ to Asp^24^ and Ser^29^ to Tyr^35^. In the ribbons model (Figure 6A,B), the possible trypsin interaction site *ku* [21], Lys^13^Gly^14^Ser^15^Ser^16^Ser^17^Thr^19^, can be recognized as an outer loop in purple.

Polarity prediction model (Figure 6C,D) and amino acid composition of arenin suggests it has few hydrophobic zones, implying a proclivity to solubilize in aqueous solutions. This is in accordance with the RP-HPLC solvent composition of 26.87% ± 0.06 acetonitrile (ACN) that prompted the elution of arenin.

## 3. Discussion

Skin anti-predator defense systems of many anurans are enriched with various types of compounds including biogenic amines, bufogenines, bufotoxins, alkaloids, peptides and proteins. Biologically active peptides of 12 to 48 amino acids have been the most studied molecules in amphibian skin secretions with over 2000 peptides classified in more than 100 families including i.e., myotropical peptides, opioid peptides, angiotensins, neuropeptides, antioxidant peptides, wound-healing peptides, antimicrobial peptides, immunomodulatory peptides, insulin-release peptide, and other peptides [8]. However, there are many reports of anurans lacking peptides in their skin secretions [11] such as the Tomato Frog *Dyscophus guineti* (Microhylidae) [22] or *Pipa carvalhoi* [10] that displayed a Kunitz-like protease inhibitor polypeptide and kynurenic acid, respectively, as major constitutive components in each species skin secretions. In the Hylidae family, at least 30 species have been suggested to lack host-defense peptides [23]. Although most amphibian skin secretions analysed through RP-HPLC display a complex profile with various peaks and similar intensities, *D. arenicolor’s* SSE reverse-phase chromatogram (Figure 2A) show few peaks with similar intensities contrasting with the number of spots with relatively similar intensity in 2D-PAGE (Figure 1). Even though the resolution difference between 2D-PAGE and RP-HPLC profiles and the amount of sample analysed by each method was largely different (150 μg of SSE for Tricine-2D-PAGE and 20 μg of SSE for analytical RP-HPLC), skin secretions from other amphibians have also been reported to present RP-HPLC profiles of reduced complexity, such as the case with *Pipa carvalhoi* [10] and *Rana tigerina* [24].

Based on our findings, skin secretions of *D. arenicolor* are highly rich in proteins with an apparent molecular weight between 20 and 37 kDa (Figure 1). However, *D. arenicolor* skin secretions do not contain any of the typical defensive 12 to 48 amino acid-long peptides described in other members of the Hylidae family. At the same time, one of the major components in the skin secretion of *D. arenicolor* is a 58 amino acid polypeptide that shares homology with anntoxin, a Kunitz-like protease inhibitor and neurotoxin first described in the skin secretions of *H. annectans*. This polypeptide that was fractionated from the SSE of *D. arenicolor* through RP-HPLC (fraction c), further resolved through Tricine-PAGE (Figure 2B), in-gel digested and analysed through mass spectrometry, showed an experimental mass between 10 and 15 kDa every time it was analysed through Tricine-PAGE. Regarding fractions d and f, even though both fractions appear as abundant as fraction c in the RP-HPLC chromatogram (Figure 2A), when total soluble protein from d and f fractions were quantified, yields were very low in contrast with fraction c. Thus, sample quantity limited the design of activity assay for both fractions. Also, when analyzed through Tricine-PAGE, fraction d showed a faint band meanwhile fraction f did not show any band at all, making difficult the in-gel digestion for MS analysis. Fraction d analysis through MS and the de novo sequencing algorithm yielded some candidate peptides with ALC > 80% that did not match with any other protein or peptide in the databases used. Although it was not possible to identify fractions d and f proteins, it is of our interest to fully characterize both fractions in future research.

Protease inhibitors (PIs) and proteases are ubiquitous molecules in nature involved in a plethora of fundamental functions. In amphibian skin secretions, PIs are known to play a key role on inhibiting the catalytic activity of proteolytic enzymes in charge of the processing of defense peptide precursors [25]. However, in amphibians lacking defensive peptides, these molecules could play a key role in immunity as a protection against extrinsic proteases produced by invading microorganisms [3]. Generally, proteases are classified based on the catalytic amino acid within their active site (aspartic acid, threonine, cysteine, serine protease) or according to the cofactor essential for catalytic activity (metalloproteases). Similarly, protein-based PIs are commonly classified based on three defined structural motifs: Kunitz, Kazal and Bowman-Birk [26]. In amphibians, Kunitz-like PIs have been found in the skin secretions of various toads, ranid and hylid frogs, Kazal inhibitors in phyllomedusinae frogs and Bowman-Birk inhibitors in ranid frogs [25]. According to the 3D model predicted for arenin (Figure 6), its structure resembles a typical Kunitz-type fold constituted by a twisted antiparallel β-sheets hairpin (residues Phe^18^-Tyr^35^) an α-helix (Leu^48^-Cys^55^), and a short 3_10_ helix (residues Tyr^3^-Glu^6^) in the N terminus [25]. Amphibian Kunitz-like PIs differ from typical Kunitz PIs in the number of disulfide bonds, since the former contains 2 disulfide bonds (Cys^5^-Cys^55^ and Cys^30^-Cys^51^ in arenin), meanwhile typical Kunitz PIs are characterized by the presence of 3 disulfide bonds [8,25].

However, even though they share 93% sequence homology, comparing the 3D model of arenin with the nuclear magnetic resonance (NMR) crystallography of anntoxin from *H. annectans*, differences in the amino acid sequence could impact arenin’s structure and, therefore, its activity. Substitution of anntoxin Gln^7^ by Glu^6^ in arenin may stabilize the short 3_10_ helix hosting the Cys^5^ residue that makes a disulfide bond with Cys^55^, this shift may provide more stability between the 3_10_ helix and the α-helix. In anntoxin, Gly^17^ is localized in the most exposed area of the reactive loop that constitutes the trypsin interaction site (*ku* domain). In arenin, Gly^17^ is substituted by Ser^16^ that may reduce the flexibility of the loop, this may decrease the selectivity of the protease inhibition activity, and at the same time, as Ser could accept or donate protons, its specific activity could be enhanced. At first glance, Asn^21^ substitution by Tyr^20^ in arenin may have little impact in overall protein stability-activity since both amino acids are polar, however the size and proximity of tyrosine’s aromatic ring to the functional groups of Phe^18^, Tyr^35^, Arg^44^ and Lys^46^, may reduce the flexibility of the reactive loop by stabilizing the twisted antiparallel β-sheets hairpin by hydrogen bonds with these 4 residues. Ser^29^ substitution by Lys^28^ may decrease protein solubility in water, since the hydroxyl group in Ser is more soluble in water than the amine in Lys.

Interestingly in PAGE (Figure 2A) analysis of the skin secretions of *D. arenicolor* and fraction c (Figure 2B), bands corresponding to arenin showed an apparent molecular weight between 10 and 15 kDa. Nonetheless, after in silico translation of its full cDNA, calculated molecular weight of the mature polypeptide based on its amino acid sequence resulted in 6578 Da. The difference between the calculated and the observed molecular weight could be explained through the formation of a homodimer between two arenin molecules, since the role of the Kunitz type domain in the formation of homodimers has been suggested previously [27]. An alternative hypothesis may be that this molecular weight shift is due to the presence of post-translational modifications such as glycosylation, methionine oxidation, tyrosine-sulfation, and/or carboxyterminal amidation. Even though these features may be found in Kunitz-like proteins from plants [28], these post-translational modifications have not been associated to amphibian Kunitz-like polypeptides. However, these features have been found in peptides and proteins from the skin secretions of other frogs [29], raising the possibility that arenin may contain them. Therefore, it is possible that arenin could be glycosylated such as the foetal human brain amyloid protein precursor that contains a Kunitz domain that has been described to present *N*- and *O*-glycosylations [30]. Yet, these hypotheses should be confirm through structural analysis of the purified protein.

At the beginning of our research we were interested in the antimicrobial potential of skin secretions of *D. arenicolor* based on ethnopharmacology studies pointing out its use in MTM activities for treatment of skin infections [16,17]. To explore this suggested antimicrobial property, *E. coli*, *S. mutans* and *S. aureus* cultures were subjected to microdilution assays to test its susceptibility against HPLC fractions and SSE at 5, 10 and 50 µg/mL. Although there is a small antimicrobial effect produced by the HPLC fractions or SSE over *E. coli*, *S. mutans* or *S. aureus*, it is not a significant response that could suggest that the main purpose of the skin secretions of *D. arenicolor* is protection against microorganisms. Moreover, the lack of classical antimicrobial peptides aids the notion that due to selective pressure, this species has developed skin secretions focused primarily, but not exclusively, on defense against non-microbial threats. This hypothesis could be supported by the demonstration of the lethal toxicity induced through the administration of high concentrations (0.05 to 3 mg/kg body weight) of anntoxin, that shares 93% homology with arenin, to potential predators such as insects, snakes, birds and mice [19]. Also, reduced or none antimicrobial activity has been observed in other Kunitz-like proteins [25].

Aside from their anti-protease activity, PIs have been associated with other underlying properties such as contribution in the termination of inflammatory processes through modulation of cytokine expression, signal transduction and tissue remodeling [26], playing a key role in the etiology and treatment of human pathologies such as cancer, inflammation and hemorrhage [25]. As a consequence of these bioactivities, PIs have been suggested as useful tools to study pathological processes along with the design of highly-specific drugs [31]. Amphibian Kunitz-like PIs have exhibited inhibition of tetrodotoxin-sensitive (TTX-S) voltage-gated sodium channel (VGSC) suggesting its application as analgesics when delivered at low concentrations [32]. While screening the effect of the skin secretions of *D. arenicolor* and arenin (fraction c) in normal human fibroblasts, colon cancer and breast cancer cells (Figure 3), a variable dose-response relationship was observed in the 3 cell lines. At low concentration of SSE and arenin (2, 4, 8 and 16 µg/mL), it is difficult to establish a trend in CaCo2 and HDFa cells; on the other hand, MCF7 cells maintain a less variable trend that is clearer with arenin alone, suggesting a role impacting cell proliferation. Based on the switches in cell proliferation caused by arenin from 2 µg/mL to 4 µg/mL and then at 8 µg/mL in HDFa and CaCo2 cells, an opposed discrete dose-dependent effect could be distinguished between these cell lines where fibroblasts viability its near to half its maximum value at the same arenin concentration (2 µg/mL) at which colon cancer cells exceeds the viability displayed by the non-treated cells. This effect is inverted at 4 µg/mL of arenin where fibroblast cells shows a viability almost the same as the non-treated cells and colon cancer cells are at its lowest viability. Once again, inversion of this effect is observed at 8 µg/mL and 16 µg/mL of arenin. At higher concentrations of arenin (32, 64 and 128 µg/mL) viability inversion is still noticeable but less drastic. This effect is not produced by SSE, since the viability of HDFa cells was lower than CaCo2 and MCF7 cells in all the concentrations tested, suggesting that skin secretions from *D. arenicolor* are more toxic to normal cells than to adenocarcinoma cells at the concentrations assessed. Overall, the response observed by the increasing concentrations of arenin and SSE on HDFa, CaCo2 and MCF7 cells could be described as an effect with high variability at low doses that is gradually stabilized into a viability reduction trend at increasing concentrations.

Hormesis is a dose-response phenomenon characterized by low-dose stimulation and high-dose inhibition, independent of biological model and endpoint, as well as chemical class and physical agent [33]. The hormetic dose-response has been shown to describe the fundamental features of several dozen receptor systems, thus affecting a vast array of biological endpoints [34], aiding the suggestion that hormetic dose-responses may represent the first comprehensive quantitative estimation of biological plasticity [35]. Some examples of systems displaying an hormetic dose-response behavior include the work presented by Bogen and collaborators [36] where human keratinocytes (HEK001) cells exposed to low-doses of arsenite (As^III^) displayed variable viability responses across the low-range of concentrations of As^III^ tested (0.25, 0.50, 1, 2, 3, 4 µM). Also, a number of cell lines displaying an hormetic dose-response when treated with a wide range of agents including antineoplastic drugs, nonneoplastic drugs, endogenous agonists and phytocompounds have been thoroughly described [37]. A molecular tactic proposed to explain hormetic dose-response relationships involves the presence of two receptors subtypes, one with high affinity and the other with low affinity for the agonist but with notably more capacity. Such an arrangement typically may lead to a biphasic dose-response, with the high-affinity receptor activated at low concentrations and the lower affinity/high-capacity receptor becoming dominant at higher concentrations [37]. Thus, it is proposed that the behavior observed in the viability of HDFa, CaCo-2 and MCF7 cells cultured in the presence of increasing concentrations of arenin and SSE displays an hormetic-like multiphasic dose-response relationship that may be regulated by more than two receptor subtypes.

Protease activated receptors (PARs) originally discovered in platelets, endothelial cells and fibroblasts, are seven-transmembrane-spanning receptors coupled to G proteins. PAR activation through protease hydrolyzation at a specific cleavage site in the extracellular N terminus of the receptor exposes a new N-terminal domain, which binds and activates the receptor initiating intracellular signals involved in responses such as platelet activation, vascular functions, inflammation, angiogenesis, neurodegeneration, proliferation, cell migration and even nociception [38,39]. Thrombin, trypsin, and human mast-cell tryptase have been found to activate the 4 PARs cloned thus far (PAR-1, PAR-2, PAR-3 and PAR-4) [40]. In theory, arenin could act as PARs antagonist through the inhibition of the proteases involved in PARs activation. This could explain the response observed at high concentration of arenin and SSE, but not the discrete effect at low concentration. It is possible that arenin could interact directly or indirectly with other cell membrane receptors additionally to PAR in a multi-targeted fashion dependent of arenin concentration according to the hormetic model hypothesis. Transcriptomic, proteomic and metabolomic analysis at different concentrations of arenin could shed light on the mechanism underlying the variable response noticed in our study.

## 4. Materials and Methods

### 4.1. Collection and Identification of D. arenicolor

Adult *D. arenicolor* of both sexes (*n* = 13, weight range 4–7 g) were collected from rocks near water bodies at Reserva de la Biosfera de Sierra Gorda, under SEMARNAT permission (SGPA/DGVS/06751/15). All specimens were kept in groups of 4 individuals in a 60 cm × 30 cm × 50 cm fish tank with soil, rocks and water, temperature in each terrarium was maintained between 20 °C to 25 °C with a heat mat, photoperiod was set to 12 h light and vitamin-supplemented live crickets were fed ad libidum. Species identification was determined through PCR amplification, sequencing and BLAST of mitochondrial DNA regions previously reported as useful markers for differential identification of *D. arenicolor* species [41]. Total genomic DNA was extracted from freeze-dried skin using the NucleoSpin^®^ Tissue kit (Machinery-Nagel, Düren, Germany). PCR was performed with ExTaq DNA Polymerase (TaKaRa Bio Inc., Kusatsu, Shiga, Japan), primers MVZ-59^F^ (5′-ATAGCACTGAAAAYGCTDAGATG-3′) and tRNAVal^R^ (5′-GGTGTAAGCGARAGCTTTKGTTAAG-3′) [42], using the following thermocycler protocol: initial denaturation of 3 min at 95 °C; 30 cycles comprised by denaturation for 10 s at 98 °C, annealing for 30 s at 53 °C, extension for 70 s at 72 °C; and a final extension for 5 min at 72 °C. Products were visualized with a 0.7% gel, purified using a Wizard SV Gel and PCR Clean-up System (Promega Corporation, Madison, WI, USA), cloned into a pGEM-T vector system (Promega Corporation, Madison, WI, USA) and sequenced at Instituto de Biotecnología de la UNAM (Cuernavaca, Mexico). All sequences obtained were subjected to online BLAST searches against Gen-Bank for identification and to check for possible contamination.

### 4.2. Recovery of Skin Secretions Extract

To promote release of skin secretions, frogs were stimulated by injecting 40 nmol/grams body weight norepinephrine bitartrate salt (Sigma-Aldrich, St. Louis, MO, USA) dissolved in sterile 200 µL water at two sites into dorsal lymph sacs. Skin secretions were collected by placing the stimulated frog in a covered glass beaker with 40 mL of collection buffer, 25 mM NaCl and 25 mM ammonium acetate, pH 7.0, [43,44] for 15 min. After removing the frog, collected skin secretions were acidified with hydrochloric acid (1% *v*/*v*) and centrifuged 30 min at 5000× *g*. Supernatants were immediately desalted and concentrated using Sep-Pak C-18 cartridges (Waters Associates, Milford, MA, USA) as previously described [18]. Concentrated and desalted protein solution was named the skin secretions extract (SSE). Protein concentration was determined using Microplate BCA^TM^ Protein Assay Kit (Thermo Scientific, Rockford, IL, USA). After norepinephrine stimulation and recovery of SSE, none of the frogs tested showed any distress related to this process. This protocol was reviewed and approved by the Institutional Committee for the Use and Care of Lab Animals of Tecnologico de Monterrey (Comité Institucional para el Cuidado y Uso de Animales de Laboratorio (CICUAL) del Tecnologico de Monterrey) under the protocol number 2015-007 (8 May 2015).

### 4.3. SSE Characterization by Reverse-Phase HPLC and 2D-PAGE

RP-HPLC profile of SSE was assessed to determine sample complexity by injecting 20 µg of skin secretions into a XBridge Peptide BEH C-18 column (4.6 mm inner diameter (i.d.) × 250 mm, 5 µm) connected to an Agilent 1200 HPLC-UV System (Agilent, Santa Clara, CA, USA). Elution was conducted at 0.75 mL/min employing solutions A (water: Trifluoroacetic acid (TFA) 99.9:0.1 *v*/*v*) and B (ACN:water:TFA 70.0:29.9:0.1, *v*/*v*/*v*) using a linear gradient from 0% to 100% solution B over 85 min. Absorbance was monitored at 214 nm and 280 nm. Absorbance spectra (190 nm to 600 nm) of most abundant peaks were analyzed as a preliminary identification step.

To evaluate the protein composition of the skin secretions, 150 µg of SSE were loaded into a 7 cm immobilized pH gradient (IPG) strip (3-10L). Isoelectric focusing (IEF) was carried out on an Ettan IPGphorTM 3 (GE Healthcare, Uppsala, Sweden). After completion of the IEF, reduction and alkylation steps as per manufacturer instructions, IPG strips were placed on home casted 10% SDS/Tricine-PAGE [45]. Second dimension electrophoresis was performed in a Mini-PROTEAN Tetra Cell (Bio-Rad, Hercules, CA, USA). Gels were stained overnight with GelCode^TM^ Blue Stain Reagent (Thermo Scientific, Rockford, IL, USA). Gels were rinsed with water several times over 6 h to remove background before being scanned in a GE Image Scanner III (GE Healthcare Bio-Sciences AB, Uppsala, Sweden)

### 4.4. Protein Isolation for Activity Evaluation

For activity screening assays to identify bioactive fractions, SEE were separated into a Zorbax SB-C18 (Agilent, Santa Clara, CA, USA) semi-preparative column (9.4 mm i.d. × 250 mm, 5 µm) connected to an Agilent 1100 HPLC-UV system (Agilent, Santa Clara, CA, USA) equipped with a fraction collector. The mobile phase system was the same as described for SSE characterization by RP-HPLC. Fractionation was performed using the following method: a linear gradient from 10% to 25% of solution B from 0 to 5 min at a flow rate of 1.5 mL/min, linear gradient to 30% of solvent B from 5 to 10 min at a flow rate of 1.5 mL/min, linear gradient to 50% of solvent B from 10 to 25 min at a flow rate of 2.5 mL/min, and a linear gradient to 100% of solvent B from 25 to 30 min at a flow rate of 3 mL/min. Total method time was 35 min. Fractions with same retention time were pooled and evaporated in a GeneVac EZ-2 series (Genevac Ltd., Ipswich, UK); protein quantification was performed as describe in the Recovery of Skin Secretions Extract section. Purity of fractions collected was assessed using HPLC and 10% SDS/Tricine-PAGE.

### 4.5. Cell Proliferation Inhibition by SSE and HPLC-Purified Fractions

To evaluate the potential effect on the inhibition of cell proliferation, SSE and HPLC-purified fractions were tested at different concentrations (2 µg/mL, 4 µg/mL, 8 µg/mL, 16 µg/mL, 32 µg/mL, 64 µg/mL and 128 µg/mL) by triplicate in two independent experiments in normal human dermal fibroblasts (HDFa, ATCC^®^ PCS-201-012), human epithelial cells from colorectal adenocarcinoma (CaCo2, ATCC^®^ HTB-37) and human epithelial cells from breast adenocarcinoma (MCF7, ATCC^®^ HTM-22). Cells were cultured in Dulbecco’s modified Eagle medium (DMEM) containing 5% fetal calf serum in flat-bottomed 96-well microtiter trays at 5 × 10^4^ cells per well at 37 °C in a humidified incubator with 5% CO_2_. After 24 h incubation, culture media was removed and fresh media containing SSE or HPLC-purified fractions was added to each well and incubated for 48 h at 37 °C in a humidified incubator with 5% CO_2_. To estimate proliferation of viable cells, 20 µL of phenazine methosulfate 3-(4,5-dimethyl thiazole-2-yl)-2,5-diphenyl tetrazolium bromide mix-based (MTS) CellTiter 96^®^ AQueous One Solution Cell Proliferation Assay (Promega Corporation, Madison, WI, USA) were added to each well and after 45 min incubation at 37 °C, absorbance at 490 nm was recorded using a 96-well plate reader. Percentage of viable treated cells was calculated in relation to untreated controls (viability percentage = treated cells Optical Density/untreated cells Optical Density × 100%) [46].

### 4.6. Statistical Analysis

Results are expressed as the mean ± standard error (SE). Statistical analysis was carried out with the statistical software JMP version 14.1.0 (SAS, Cary, NC, USA). One way-analysis of variance (ANOVA) was used to analyze the variation between groups. Tukey’s test was used to identify significantly different means. Results were considered significant if *p* ≤ 0.05. Significantly different means are identified with different significance letters.

### 4.7. Structural Characterization of Bioactive Protein

#### 4.7.1. In Gel Digestion of Bioactive Proteins

Isolated fractions were solubilized in sodium dodecyl sulfate (SDS) and resolved in a 12% Tricine-SDS-PAGE. Gel was stained with Colloidal Coomassie Blue and protein bands were cut and destained in 50% ACN containing 25 mM ammonium bicarbonate (ABB).

In gel digestion of proteins was performed as previously described [47], with minor modifications. Briefly, gel bands were cut in 1-mm^2^ cubes and proteins were reduced in 10 mM dithiothreitol in 25 mM ABB at 60 °C for 1 h. Proteins were subsequently alkylated with 55 mM iodoacetamide in 25 mM ABB at room temperature for 45 min protected from the light. Gel cubes were then dehydrated with ACN in two washes of 1 min/each and dried during 5 min in the speedvac. Tryptic digestion of proteins was performed at 37 °C overnight by the addition of 10 ng/µL of sequencing grade trypsin prepared in 25 mM ABB. Tryptic digested peptides were extracted from the gel cubes twice with 50% ACN, 5% acetic acid during 15 min with vigorous vortexing. Subsequently, two rounds of extraction were performed with 50% ACN, 5% formic acid (FA) for 15 min/each. The obtained supernatants were combined and dried in the speedvac. Dried peptides were resuspended in 3% ACN, 0.1% FA for their subsequent analysis by liquid chromatography mass spectrometry.

#### 4.7.2. Liquid Chromatography Mass Spectrometry (LC-MS) Analysis of Bioactive Proteins

Primary structure of the bioactive proteins was characterized by MS as previously described [48,49] with minor modifications. Analysis of peptides was performed using a QExactive mass spectrometers coupled with a Dionex UltiMate 3000 UHPLC system from Thermo Fisher Scientific Inc. (Bremen, Germany). Peptide separation was performed using a reverse-phase EASY-Spray LC column (75 μm ID × 15 cm, 3 μm particle size, Thermo Scientific Inc., Bremen, Germany) maintained at 35 °C and working at 300 nL/min. Eluents A (0.1% FA) and B (90% ACN, 0.1% FA) were used to establish the following 60-min gradient: 7–18% B for 30 min, 18–32% B for 15 min, 32–50% B for 4 min, 50–90% B for 1 min, 90–5% B for 0.1 min, 5% B for 7.9 min, 5–7% B for 2 min. Spray was generated using a Thermo Scientific EASY-Spray source (Bremen, Germany) at 1.75 kV. QExactive mass spectrometer was set to positive mode for data acquisition with Xcalibur 3.0.63 software (Thermo Fisher Scientific Inc., Bremen, Germany) software alternating between full Fourier transform-mass spectrometry (FT-MS) (350−1600 *m*/*z*, resolution 75,000, with 1 μscan per spectrum) and FT-MS/MS (resolution 35000, with 1 μscan per spectrum). Fragmentation of the 10 most intense precursors with charge > +2 and isolated within a 2 Da window was performed using a normalized collision energy of 28%. A threshold of 500 counts was enabled. For full FT-MS and FT-MS/MS automatic gain control was set to 3 × 10^6^ and 2 × 10^5^, respectively.

#### 4.7.3. Bioinformatics and Data Analysis 

The raw MS/MS data were processed using PEAKS software version 7.5 (Bioinformatics Solutions, Waterloo, ON, Canada) for the de novo sequencing of peptides [50]. In parallel data were searched using PEAKS software against a tailored database containing all anuran mRNA (648,884) and protein (425,093) entries available in the National Center for Biotechnology Information (NCBI, https://www.ncbi.nlm.nih.gov/). For the de novo sequencing of peptides, local confidence was considered as the confidence (%) that a particular amino acid was present in the de novo peptide at a particular position. The sum of the total confidence scores (0 to 1) from each amino acid in the peptide sequence divided by the number of amino acids is presented to be ALC used to assess the accuracy of the interpretation. The database search was performed as previously described [51], allowing a precursor ion tolerance of 10 ppm, a fragment tolerance of 0.05 Da MS/MS and a false discovery rate of 1% at peptide level. Carbamidomethylation at Cys residues was set as a fixed modification.

### 4.8. Identified Protein cDNA Synthesis

To identify the DNA sequence codifying for the protein analyzed by MS, cDNA synthesis was performed using mRNA isolated from skin secretions as template for retro-transcription. After neuroendocrine stimulation, skin secretions were recovered by directly rubbing the stimulated skin with a sterile tip and immediately transferred into a sterile polypropylene tube containing 1 mL of lysis/binding buffer provided by the Dynabeads^®^ mRNA purification kit (Ambion, Carlsbad, CA, USA). Polyadenylated mRNA was isolated as per manufacturer’s instructions.

First strand cDNA synthesis was carried out using the 3′RACE CDS Primer A from SMARTer RACE 5′/3′ kit (Clontech, Mountain View, CA, USA) and the Improm-II Reverse Transcriptase (Promega Corporation, Madison, WI, USA). 3′-RACE PCR was performed using a universal primer mix (UPM), supplied with the SMARTer RACE 5′/3′ kit, a degenerated sense primer (RAnx1: 5′-GAARACWTCTGTKGTKTTYYTGG-3′) and high fidelity polymerase TaKaRa Ex Taq (Clontech, Mountain View, CA, USA) with the following program: initial denaturation step: 60 s at 94 °C; 35 cycles: denaturation 30 s at 94 °C, primer annealing for 30 s at 56 °C, extension for 180 s at 72 °C. The resulting PCR fragments (378 bp) were purified with a Wizard SV Gel and PCR Clean-Up System (Promega Corporation, Madison, WI, USA), cloned using a pGEM-T vector system (Promega Corporation, Madison, WI, USA) and sequenced at Instituto de Biotecnología de la UNAM (Cuernavaca, Mexico). All sequences obtained were subjected to online BLAST (Available online: https://blast.ncbi.nlm.nih.gov/Blast.cgi) searches against GenBank for identification and to check for possible contamination. cDNA was confirmed by in silico translation of sequenced PCR products and further alignment against digested peptides with highest ALC. Signal peptide of the sequenced cDNA was predicted in the SignalP 4.1 server (Available online: http://www.cbs.dtu.dk/services/SignalP/) [52].

### 4.9. Structural Modeling of Arenin

After confirmation of the correlation between the cDNA generated with the mRNA recovered from the skin secretions of *D. arenicolor* and the MS analysis performed to fraction’s c protein, a 3D model of the in silico translated protein was generated using the SWISS MODEL server (Available online: https://swissmodel.expasy.org/) [53,54]. Protein model generated was downloaded and modified with the molecular visualization software UCSF Chimera (RBVI, San Francisco, CA, USA) [55]. A structural comparison between the identified *D. arenicolor*’s skin secreted protein and anntoxin, the protein used as a template for modeling (PDB: 2kcr) [19], was performed in order to detect conformational differences.

## 5. Conclusions

The current work demonstrates the lack of classical defensive peptides in the skin secretions of *D. arenicolor*. At the same time, we present the full cDNA of the third gene-encoded anntoxin-like protein from amphibian skin secretions, arenin. Based on the cell proliferation assay and the homology between arenin and the Kunitz-type protease inhibitor anntoxin, arenin is proposed to induce a hormetic-like dose-dependent behavior in the proliferation of normal and cancerous cells, partially through the protection of PARs. Nonetheless, further research focused on transcriptomics, metabolomics and proteomics changes is needed in order to confirm this hypothesis and to identify other receptors and routes involved in order to elucidate the mechanism of action behind the dose-response relationship observed in HDFa, CaCo2 and MCF7 cells cultured with low concentrations of arenin.

## Abbreviations

ABB	Ammonium bicarbonate
ACN	Acetonitrile
ALC	Average local confidence
BPTI	Bovine pancreatic trypsin inhibitor
FA	Formic Acid
FT-MS	Fourier transform-mass spectrometry
IEF	Isoelectric focusing
IPG	Immobilized pH gradient
MS	Mass spectrometry
MTM	Mexican Traditional Medicine
MTS	Phenazine methosulfate 3-(4,5-dimethyl thiazole-2-yl)-2,5-diphenyl tetrazolium bromide mix-based
PAR	Protease activated receptor
PIs	Protease inhibitors
SDS	Sodium dodecyl sulfate
SSE	Skin secretions extract
TEAB	Triethylammonium bicarbonate
TFA	Trifluoroacetic acid
TTX-S	Tetrodotoxin-sensitive
VGSC	Voltage-gated sodium channel
